# Dual-task gait speed and mobility are positively associated with declarative memory

**DOI:** 10.1093/geroni/igab046.3180

**Published:** 2021-12-17

**Authors:** Michelle Gray, Sally Paulson, Joshua Gills, Erica Madero, Jennifer Myers, Anthony Campitelli, Megan Jones, Jordan Glenn

**Affiliations:** 1 University of Arkansas at Fayetteville, Fayetteville, Arkansas, United States; 2 University of Arkansas at Fayetteville, Cincinnati, Ohio, United States; 3 Neurotrack Technologies, Redwood City, California, United States

## Abstract

In the US, it is not recommended to perform routine screening assessments for cognitive function or impairment among older adults, due to the lack of effective pharmacological treatments. These common practices result in delayed identification and treatments for slowing cognitive decline progression. Thus, the purpose of the present investigation was to determine the ability to predict cognition from common measures of physical function. Seventy-five community-dwelling older adults (80.7±5.4 years) completed physical function and cognitive assessments. Physical function was assessed using the Short Physical Performance Battery (SPPB), peak velocity during a power sit-to-stand task, and dual-task walking test. Cognition (declarative memory) was assessed using a validated Visual Paired Comparison test. 38% of the variance in cognition was accounted for by the predictor variables (age, sex, education, SPPB, dual-task, peak velocity). Significant predictors included dual-task walking (p = .03), SPPB (p = .02), and education (p = .02). For each 1 second faster during the dual-task performance, cognition increased by 4 percentile units. Likewise, each 1 unit increase in SPPB resulted in an increase of 4 percentile points in cognition. The results indicate more than a third of the variance in declarative memory can be predicted by commonly assessed measures of physical function. This information is useful when identifying older adults that may have cognitive impairment before overt signs are realized. With the lack of recommended cognitive testing, using physical function declines to identify possible cognitive decline is promising. These results are preliminary in nature and longitudinal determination is warranted.

